# OUTCOMES OF A PALLIATIVE CARE INTERVENTION FOR ADULTS WITH DEMENTIA IN SKILLED NURSING FACILITIES

**DOI:** 10.1093/geroni/igae098.4038

**Published:** 2024-12-31

**Authors:** Elisha Oduro, Joan Carpenter, Nancy Hodgson, Laura Hanson, Shijun Zhu, Merve Gurlu, Mary Ersek

**Affiliations:** University of Maryland Baltimore, Baltimore, Maryland, United States; University of Maryland Baltimore, Baltimore, Maryland, United States; University of Pennsylvania, Philadelphia, Pennsylvania, United States; University of North Carolina School of Medicine, Chapel Hill, North Carolina, United States; University of Maryland School of Nursing, Baltimore, Maryland, United States; University of Maryland Baltimore, Baltimore, Maryland, United States; Department of Veterans Affairs, Philadelphia, Pennsylvania, United States

## Abstract

Older adults living with Alzheimer’s disease and related dementias (ADRD) often receive disease-focused rehabilitative care in post-acute skilled nursing facilities (SNF) without access to palliative care (PC). To address this gap, our team developed a primary PC intervention and tested it among SNF patients with ADRD. We conducted a pilot pragmatic clinical trial comparing the primary PC intervention to usual care in 9 SNFs in the MidAtlantic US. Using a nonequivalent group design, SNF resident participants (n=18) were clustered by site and assigned to intervention or control group in a 2:1 (respectively) ratio. We measured patient quality of life with the Palliative Outcome Scale Version 2 (POSv2, range 0-40; higher scores reflect poorer quality of life) and analyzed data using linear mixed effects model. Patients (n=13 intervention, n=5 control) had a mean age 84 years, were mostly White (94%) and female (72%). Our results showed that quality of life had a trend of improvement for the intervention group (POSv2 baseline=12, follow up=10.5) but not for the control group (POSv2 baseline=6.6, follow up=9.3); the intervention demonstrated a clinically meaningful effect yet was not statistically significant (effect size=-.51, p=.20). Our findings suggest that a primary PC intervention is promising in its effect to improve quality of life among patients with ADRD during SNF care. Future work will focus on testing the intervention in a larger more diverse sample and exploring the most valuable intervention components as assessed by patients with ADRD and their care partners.

